# Validation of the three-chamber strategy for studying mate choice in medaka

**DOI:** 10.1371/journal.pone.0259741

**Published:** 2021-11-15

**Authors:** Ena Kaneko, Hinako Sato, Shoji Fukamachi

**Affiliations:** Department of Chemical and Biological Sciences, Laboratory of Evolutionary Genetics, Japan Women’s University, Bunkyo-ku, Tokyo, Japan; Imperial College London, UNITED KINGDOM

## Abstract

The three-chamber experiment, in which one test animal can choose between two animals placed in physically inaccessible compartments, is a widely adopted strategy for studying sexual preference in animals. Medaka, a small freshwater teleost, is an emerging model for dissecting the neurological/physiological mechanisms underlying mate choice for which intriguing findings have been accumulating. The three-chamber strategy has rarely been adopted in this species; therefore, here we investigated its validity using medaka colour variants that mate assortatively. First, a total of 551 movies, in which a test male and two choice females interacted for 30 min under a free-swimming condition, were manually analysed. The sexual preference of the males, calculated as a courtship ratio, was highly consistent between human observers (*r* > 0.96), supporting the objectivity of this manual-counting strategy. Second, we tested two types of three-chamber apparatuses, in which choice fish were presented in either a face-to-face or side-by-side location. Test fish (regardless of sex) spent most of the time associating with choice fish in the compartments. However, their sexual preference, calculated as an association ratio, was poorly reproduced when the locations of the choice fish were swapped. Third, the sexual preferences of males quantified using the manual-counting and either of the three-chamber strategies did not correlate (*r* = 0.147 or 0.297). Hence, we concluded that, even for individuals of a species like medaka, which spawn every day, sexual preference could not be reliably evaluated using the three-chamber strategy. Optimization of the protocol may solve this problem; however, the explanation for the observation that animals that are ready for spawning persist with never-accessible mating partners must be reconsidered.

## Introduction

**Fig 1:**
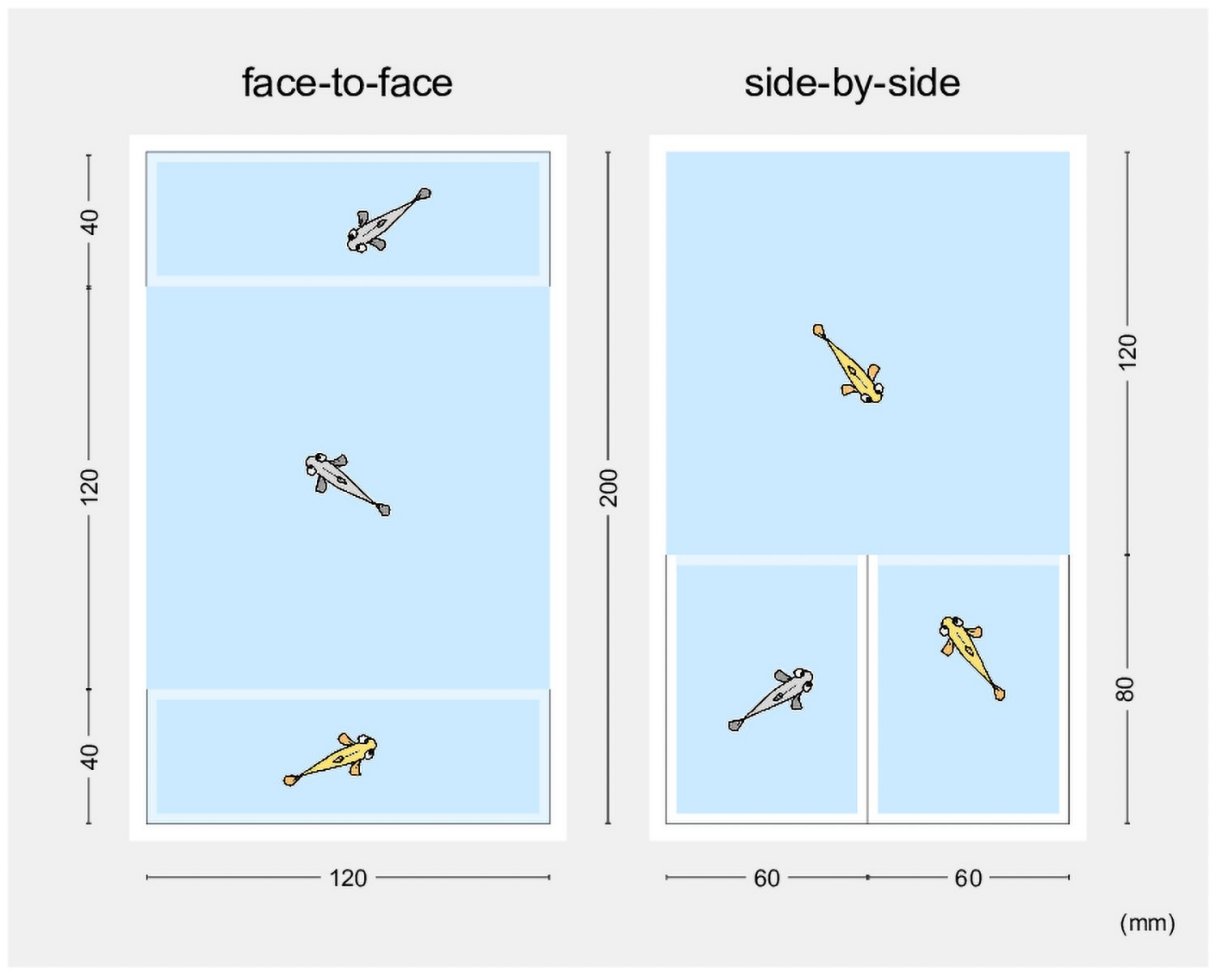
Diagram of the three-chamber apparatuses examined in this study. Two small cases (external dimensions of W120 × D40 mm or W60 × D80 mm) were placed in a large tank (internal dimensions of W120 × D200 mm) as choice compartments in a face-to-face (left) or side-by-side (right) orientation. Each case had a bottom and there was no water flow between any of the compartments (i.e., the visual and mechanical, but not olfactory, cues of the choice fish were available for the test fish). Opaque (white) or transparent walls are illustrated as white or light-blue lines, respectively. The water level was about 5 cm from the bottom in all the compartments. The outer white tank was also used for the manual-counting experiments, without the small cases.

Mate choice in animals has been attracting the attention of zoologists as a research subject. The choice is largely made by the female in many species, which requires the male to exaggerate himself (e.g., peacock) or defeat his rivals (e.g., lion) to be chosen. Nevertheless, there are many cases in which mate choice lies with males [1]. The sexual preference of males and females is often investigated via experiments using a three-chamber apparatus (Fig 1). A tank or cage is partitioned into three compartments by transparent walls or wired nettings. Two choice animals (i.e., animals to be chosen) are then placed in two of the compartments typically located on both ends of the apparatus in a face-to-face manner (Fig 1, left) or on either end in a side-by-side manner (Fig 1, right) and the test animal is placed in the remaining compartment, where its behaviour is analysed. The preference of the test animal is quantified according to the time of association with the choice animals. Moreover, the number of courtships (e.g., dances or songs) are additionally counted in some studies. Such studies unveiled significant association preferences in many species, including vertebrates and arthropods. The choice animals could be replaced by digital images, and many intriguing results were obtained by modifying the size, shape, colour or motion of the images [2–5].

Medaka (*Oryzias latipes*) is small freshwater fish that is native to the Far East and has been used in research for more than a century [6] (see also; https://genestogenomes.org/100-years-since-the-medakas-international-debut-aidas-legacy/). A growing number of articles have reported the mating behaviour of medaka, as well as its molecular/cellular background. For instance, females hesitate to mate with males with which they are not visually acquainted, a behaviour that depends on gonadotropin-releasing hormone 3 neurons or oxytocin [7, 8]. Moreover, males repel their rivals away from their females, which is a mate-guarding behaviour that requires vasopressin [9]. A bright-orange variant with a mutation on the *solute carrier family 45 member 2* gene is not discriminated from a dark-brown wild-type fish as a mating partner [10]. A lack of the oestrogen receptor 2b leads females to display male-typical courtship behaviours without abolishing oogenesis [11]. Somatolactin alpha (SLα), which controls body colour, also build a pre-mating barrier between sexually compatible strains by affecting mating preference [12]. This sexual isolation is relaxed under monochromatic lighting [13], when one of its *cone-opsin* genes is knocked out [14, 15] or when the colour variants are reared in a mixed manner from hatching [16].

An outstanding feature of medaka that renders it an excellent model for studying mate choice is that it mates every day. If these fish are kept under appropriate conditions (i.e., 14-h lighting and a water temperature of 25°C with sufficient food), researchers can observe their mating behaviours, including actual spawning, every morning. Hence, the sexual preference of female medaka can easily and unmistakably be qualified by identifying the males with which they mated. Their sexual preference can also be quantified by measuring the time (latency) until spawning [8–10, 12] (but only when it is guaranteed that all males approach any female equally).

**Fig 2:**
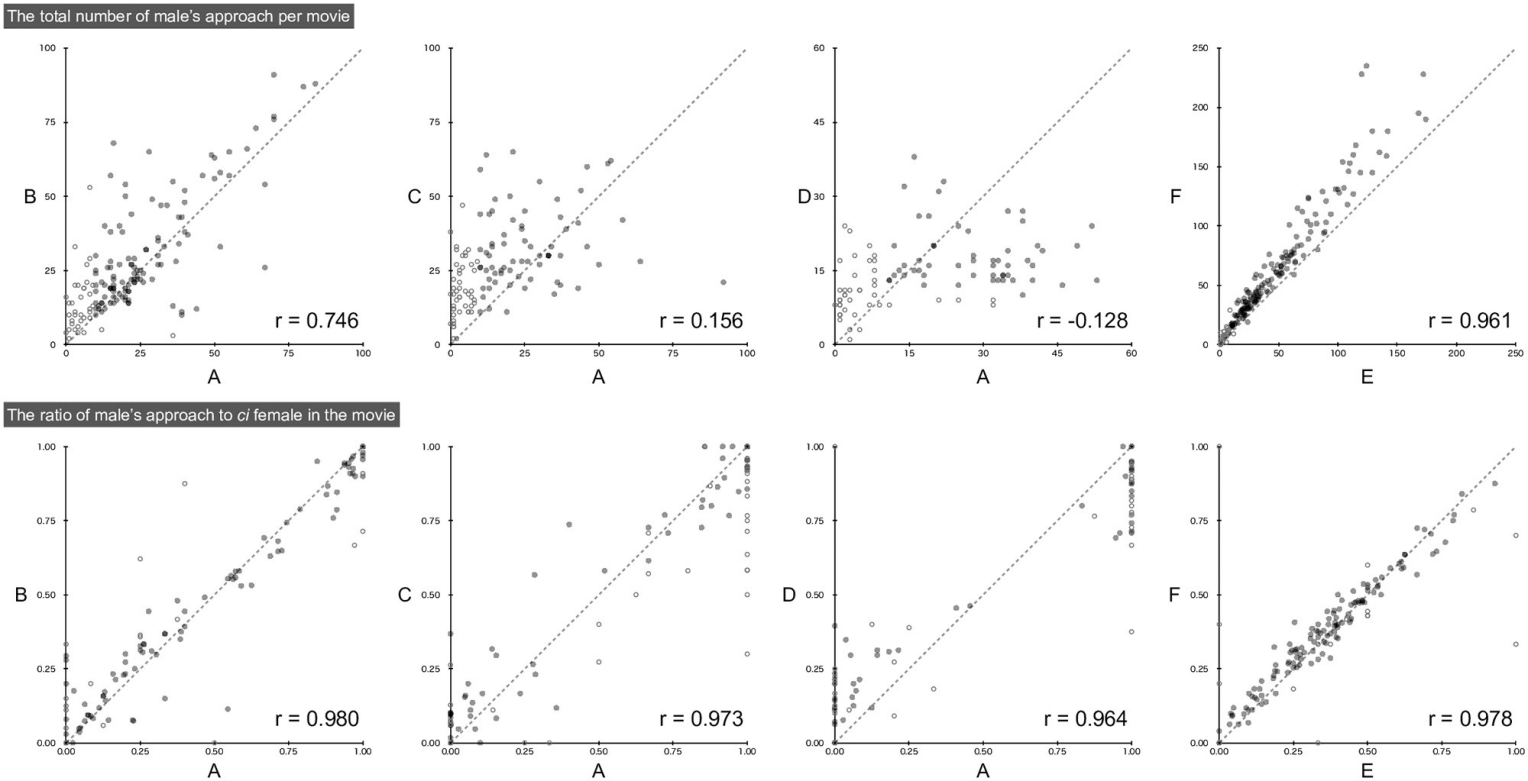
Quantification of the sexual preference of males using the manual-counting strategy. Top: comparison of the total number of approaches by the male per movie between two out of six observers (A–F). The dotted line shows y = x (i.e., the number was identical between the observers). Each dot (white or grey) represents a result from one movie, but we excluded white dots (indicating that less than 10 approaches were detected by either/both observer/s) from the calculation of the correlation coefficient shown on the bottom right of each graph. Bottom: comparison of the ratio of the approaches directed to the *ci* female (i.e., sexual preference of the males). Each dot represents one movie. Note the strong correlations in ratio (bottom), despite the low or absent correlations in number (top).

The sexual preference of medaka males must be qualified/quantified differently because whether or not he can finally spawn depends on the female. Although males can spawn 17 times per morning on average, when given a chance [17], females often ignore or even escape from approaching males and do not let them spawn. This problem (i.e., a male’s choice can easily be delayed/invalidated by a female’s rejection) could be solved by focusing on the initial, rather than the eventual mating behaviour; i.e., the approaching rather than the spawning. A similar strategy is adopted in fish that undergo internal fertilization, in which thrusts of the gonopodium or copulation attempts can be counted manually [18, 19]. The approaching behaviour of medaka males is apparent and occurs very frequently (e.g., more than 200 times in 30 min; see Fig 2), regardless of whether the females have already spawned (females sometimes spawn multiple times per morning [12]). Thus, by presenting two choice females to one test male under a free-swimming condition, his sexual preference can be qualified/quantified by counting his approaches manually [10, 12–16].

Although this manual-counting strategy has been effective, the definition of “approaching” (i.e., a male’s quick movement toward a female to position beneath her) is somewhat subjective and should vary among human observers. More objective criteria, such as the association time in the three-chamber experiment, would be preferable from a scientific point of view. In addition, the manual detection of an approach event within a split second requires a high frame-per-second (fps) rate of video recording and full concentration during the analyses. If the three-chamber strategy can be applied, the positions (x–y coordinates) of test fish could be extracted automatically from time-lapse images (e.g., 1 fps), which would greatly reduce the file size and simplify the analyses (as experienced in this study).

Thus, the purpose of the present study was to investigate whether the face-to-face or side-by-side three-chamber strategy could be applied for assessing the sexual preference of medaka males. For this purpose, we quantified the sexual preference of two colour variants, *color interfere* (*ci*) and Actb-SLα:GFP, using the free-swimming and three-chamber strategies, and compared the results. These colour variants strongly prefer to mate within a strain (i.e., they are sexually isolated), but this preference could artificially be weakened to various degrees by modifying the rearing conditions or *cone-opsin* repertoires [14–16].

## Materials and methods

### Ethics statement

The animal study was reviewed and approved by the Animal Experiment Committee of Japan Women’s University.

### Fish

Two body-colour variants of medaka, the pale-grey *ci* and the dark-orange Actb-SLα:GFP strains [20, 21], were used here (see Fig 7 for their body colours). They hatched and grew in our laboratory in which filtrated water that was kept at a temperature of 25°C, with light provided by white LED bulbs for 14 h (08h30 to 22h30) per day. Larvae and adults were fed five times a day between 10h00 and 18h00 with live brine shrimp and commercial flake food.

When the *ci* and Actb-SLα:GFP strains are reared separately since hatching, males have little sexual interest in the females of the other strain [12, 16]. To include fish with a weakened sexual preference, we included *ci* and Actb-SLα:GFP individuals that had been reared in a mixed manner for various periods since hatching or in which the *blue-opsin* (*short-wavelength sensitive 2*; *SWS2*) gene had been knocked out (i.e., the *sws2*^*+14b*^ and *sws2*^*+1a+14b*^ lines in the *ci* or Actb-SL:GFP genomic background [15]). All of these fish with various degrees of sexual preference are referred to as “*ci*” and “Actb-SLα:GFP” without discrimination in this article.

### Mate-choice assessment via manual counting

On the day before the experiments, males and females were separated using a translucent divider with slits, to prevent spawning. On the following morning, one test male (either *ci* or Actb-SLα:GFP) and two size-matched (less than ±1 mm in body length) choice females (one *ci* and one Actb-SLα:GFP) were placed in a white acrylic tank (with the following interior dimensions: width, 120 mm; depth, 200 mm; Fig 1) and their mating behaviour was video recorded for 30 min. Subsequently, another mate-choice experiment was performed after replacing the male with a male of the other strain. On the following day, the same males were used for the second mate-choice experiments, in which a different pair of *ci* and Actb-SLα:GFP females was presented. We often repeated the experiment an additional two times. Thus, we recorded two or four movies (a total of 60 or 120 min) for each male by presenting two or four pairs of females on two or four consecutive days.

By manually observing the movies, we counted the number of male approaches to both females and quantified the sexual preference of the males as a ratio of the approaches to the *ci* female; i.e., >50% or <50% implying that the male preferred the *ci* or Actb-SLα:GFP female, respectively. We discarded results in which less than 10 approaches could be counted in a movie.

### Mate-choice experiment using a three-chamber apparatus

Two types of three-chamber apparatuses were constructed by placing two all-side or one-side transparent cases (exterior dimensions of W120 × D40 mm or W60 × D80 mm) into the white tank described above in a face-to-face or side-by-side orientation, respectively (Fig 1). We used the one-side (instead of all-side) transparent cases to avoid visual contact between neighbouring choice fish in the side-by-side location. In both orientations, the third compartment (i.e., outside the small cases but inside the white tank) into which a test fish was placed became W120 × D120 mm (interior dimensions). The compartments were physically separated and there was no water flow between them (i.e., no odour contact between test and choice [or choice and choice] fish).

Males and females, which had been separated by the divider on the day before the mate-choice experiments, were placed in the compartments (two size-matched choice fish in the cases, and one test fish in the tank), and the positions of the test fish were recorded as time-lapse images at 1 s intervals for 30 min. Subsequently, another mate-choice experiment was performed using the same three fish but using a swapped location of the choice fish. Thus, we video recorded each test fish for a total of 60 min while presenting one pair of choice fish in different locations on the same day.

The position (x–y coordinate) of the test fish in each time-lapse image was extracted using UMATracker software [22]. We virtually divided the test compartment into 10 × 10 = 100 areas (W12 × D12 mm each) and considered that a test fish was associating with either of the choice fish when its x–y coordinate was located within 12 or 24 mm from the choice compartments in the face-to-face or side-by-side apparatus, respectively (note that this definition rendered the non-association [neutral] zone W120 × D 96 mm in both apparatuses). The sexual preference of the test fish was calculated as a ratio of the association with *ci* (i.e., >50% or <50% meaning that the test fish preferred the *ci* or Actb-SLα:GFP individual, respectively). We discarded results in which the total association time of the test fish was <10 min (i.e., 600 time-lapse images).

### Correlation analysis

To evaluate the reproducibility of the manual-counting approach between human observers, we performed a correlation analysis using the Numbers software (Apple). Although males were repeatedly used for preparing these movies, we regarded that all data were independent because their repeated use should not affect the evaluation of the consistency between humans. To evaluate the reproducibility between the manual-counting and three-chamber strategies, we also regarded all data as being independent because each datum was calculated as the average of each male.

## Results

### The manual-counting strategy yielded objective results

We placed one test male (*ci* or Actb-SLα:GFP; 148 fish in total) and two size-matched choice females (*ci* and Actb-SLα:GFP; 152 fish in total) in a white tank (W120 × D200 mm, with a water level of about 5 cm; Fig 1) and video recorded their mating behaviours for 30 min. A total of 551 movies (each male was used two or more [basically four] times and presented with different pairs of females) were doubly analysed by two of six human observers (A–F). Observers A and B, A and C, A and D, and E and F analysed 152, 118, 96, and 185 movies, respectively.

The total number of male approaches per movie ranged from zero to 235, reflecting various degrees of enthusiasm among the males regarding mating on the day of the experiment. Analyses of an identical movie did not necessarily yield an identical number of total approaches between the observers (Fig 2, top). Nevertheless, the number was highly correlated at least in one comparison (*r* = 0.961 for E vs. F), although F always detected approaches more sensitively than E did (i.e., their definition of “approach” seemed to be different). In other comparisons, the correlation was low or even absent (e.g., *r* = −0.128 for A vs. D). Various explanations for these results were considered; e.g., inconsistent concentration during movie analyses by either or both observers, vague definition of “approach” or any type of descriptive error. Thus, we concluded that the total number of male approaches is an observer-dependent and subjective parameter.

Nevertheless, the ratio of approaches (the percentage of approaches to the *ci* female) was strongly correlated between observers in all cases (*r* = 0.964–0.980; Fig 2, bottom). Therefore, as long as the male’s approach was detected based on an equal definition (i.e., sensitively or insensitively) per movie for both females, the ratio (i.e., sexual preference) could consistently be quantified by any of the observers. It should also be noted that many of the data that were regarded as being invalid because less than 10 approaches were recorded (shown as white dots in Fig 2) were plotted close to the y = x lines, indicating that this criterion might unnecessarily reduce the sample size. Taken together, these results revealed that, as long as the sexual preference was quantified as a ratio, the manual-counting strategy could be minimally subjective, if not objective, and that the ratio could be regarded as a reliable parameter.

### The test fish had a strong interest in the choice fish in the three-chamber environments

**Fig 3:**
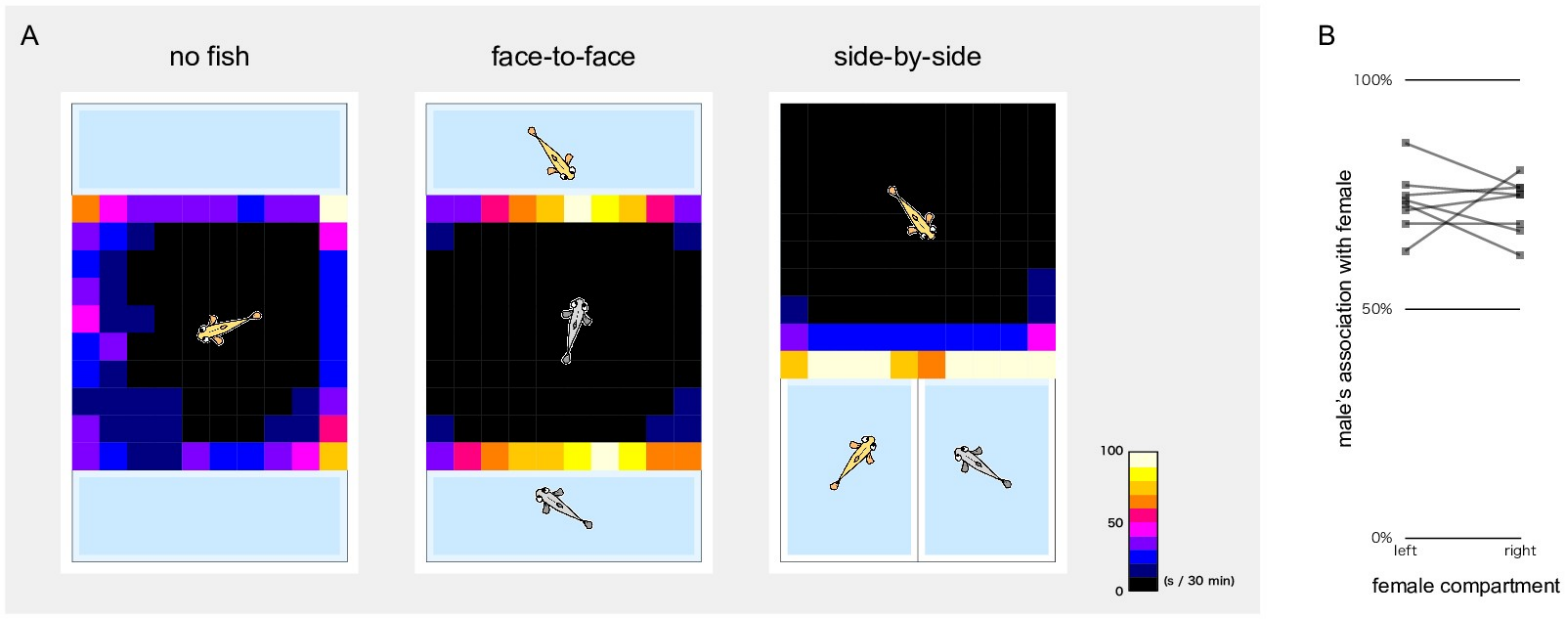
Position of test fish during the three-chamber experiments. (A) Heatmaps showing the averaged positions of test males (from the left, n = 8, 32 and 20). In the face-to-face orientation (left and middle), males spent most of the time in front of the choice compartments, but only when females were placed inside them. In the side-by-side three-chamber apparatus (right), males also spent most of the time in front of the choice compartments. Based on these heatmaps, we defined “association zones” to be within 12 or 24 mm from the choice compartments in the face-to-face or side-by-side orientation, respectively. This definition rendered the area of neutral (not-associating) zone W120 × D96 mm in both apparatuses. (B) Side-by-side three-chamber experiments with an empty compartment. Test males (n = 8) were given only one choice female in either the left or right compartment, and the results of the same male are connected by the grey line. All males always preferred to associate with the females.

In the face-to-face three-chamber apparatus (Fig 1, left), we tested 32 males (20 *ci* and 12 Actb-SLα:GFP individuals) and performed two experiments per male with swapped female locations. The males were located within 12 mm from choice compartments (i.e., associated with females) for 74.8% ± 2.4% (mean ± SEM) of the 30 min observation period (Fig 3A, middle). When the choice compartments were emptied, males (n = 8, four each of the *ci* and Actb-SLα:GFP backgrounds; one experiment per male) tended to be at the corner or alongside the wall of the test compartment (Fig 3A, left). Thus, medaka males had an apparent and persistent interest in the females, rather than the empty spaces behind, or their own images potentially reflected on, the transparent walls.

In the side-by-side apparatus, we analysed 20 males (nine *ci* and 11 Actb-SLα:GFP individuals; two experiments per male with swapped female locations). The males showed a strong and persistent interest in females and spent 85.4% ± 2.9% of the 30 min observation period within 24 mm from the choice compartments (Fig 3A, right). When either of the compartments was emptied, the males (n = 8 Actb-SLα:GFP individuals; two experiments per male with swapped female locations) always spent more time in front of a compartment with an Actb-SLα:GFP female (72.9% ± 1.6% of the total association time; Fig 3B), further supporting the contention that the males were not interested in the empty compartments or transparent walls, but in the females.

### Validation of the face-to-face three-chamber strategy

**Fig 4:**
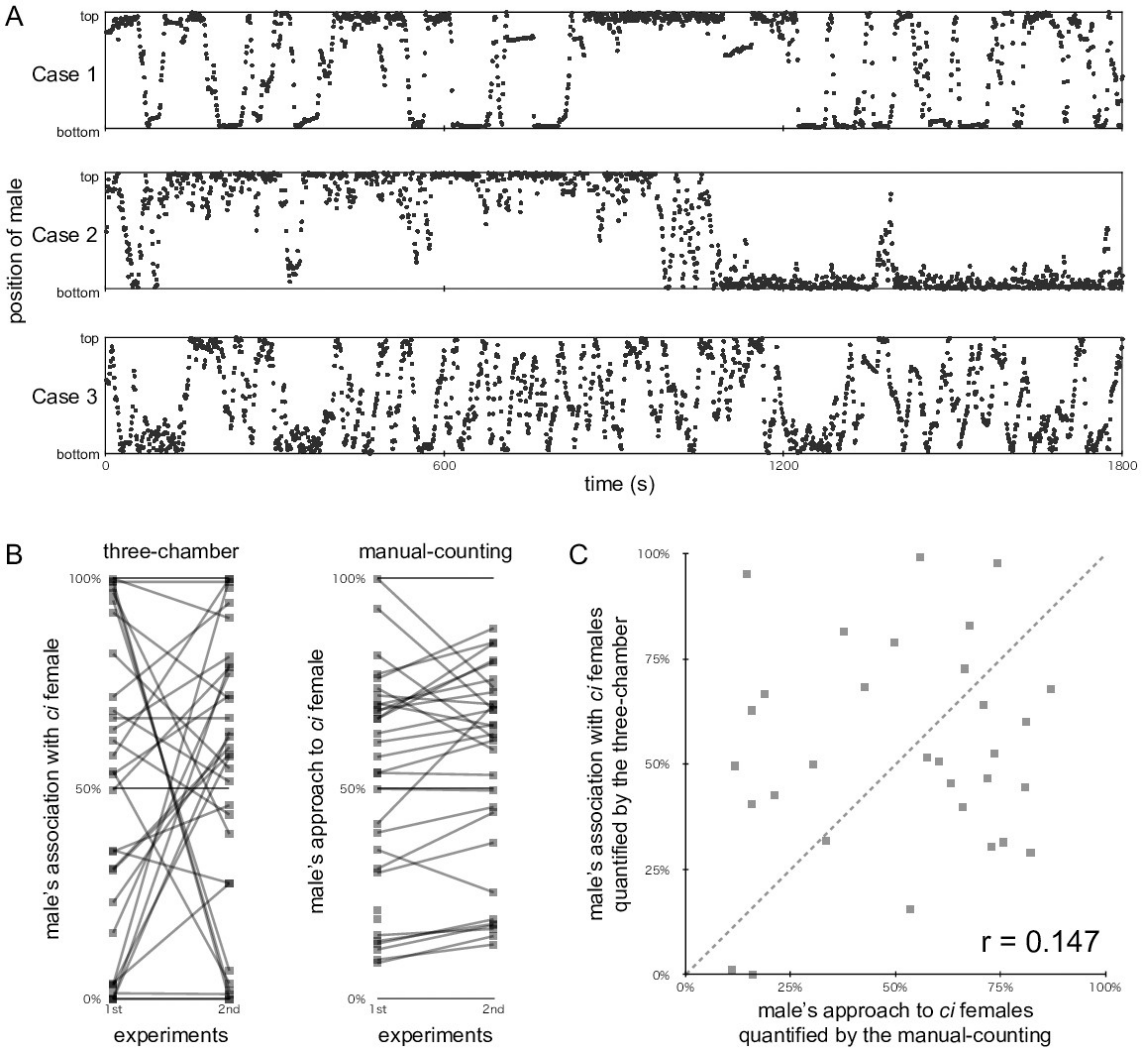
Assessment of the face-to-face three-chamber strategy. (A) Examples of the movement of test males between choice compartments (top and bottom). The positions of a male were plotted at 1 s intervals during the 1,800-s experiment. Males in Cases 1 and 2 showed interest in both females, but their movements were different; i.e., the female with which the male associated was frequently or rarely changed in Cases 1 or 2, respectively. The male in Case 3 was located in the association zones for less than 10 min; therefore, this datum was excluded from subsequent analyses. (B) Reproducibility of the preference of males in the three-chamber (left) or manual-counting (right) experiments. Test males (n = 32) were analysed using both the three-chamber and manual-counting strategies (i.e., each male was used in four experiments). Results (shown as squares) from the same male are connected by the grey line. Results that did not fulfil our criteria (i.e., at least a 10-min association or 10 approaches in the three-chamber or manual-counting experiments, respectively) were not shown in these graphs (thus, a few squares remained unconnected). Note that many grey lines crossed the horizontal 50% line in the three-chamber experiment, meaning that the male preference detected in the first experiment was reversed in the second experiment. Such crossing grey lines were seldom found in the manual-counting experiment. (C) Correlation analysis of the three-chamber and manual-counting results shown in (B). The preferences quantified in the first and second experiments were averaged per male and plotted in the graph. The dotted line indicates a y = x line.

The typical behaviours of test males during the face-to-face three-chamber experiments are exemplified in Fig 4A. In Cases 1 and 2, the males showed interest in both choice females (referred to as top and bottom) and spent a similar amount of time in front of them (57.9% vs. 42.1% and 50.3% vs. 49.7%, respectively). However, their behaviours were different in that the male in Case 1 frequently changed his association targets, whereas the male in Case 2 basically did so only once (at about 17 min). Case 3 was one of three (out of 64) results that was judged to be invalid, because this male associated with females for less than 10 min (namely, 8.6 min).

From 0% to 100%, various ratios of association with the *ci* female were detected in the face-to-face experiments, but these preferences were often reversed when the location of choice females was swapped (Fig 4B, left). When the sexual preference of these males (n = 32) was assessed using the manual-counting strategy, the results were largely reproducible between the first and second experiments (Fig 4B, right). It should be noted that different pairs of females were presented on different days in the manual-counting experiments, whereas an identical pair was presented (in swapped locations) on the same day in the three-chamber experiments. We averaged the results of the first and second experiments for each male and assessed the correlation between the manual-counting and three-chamber results. As shown in Fig 4C, this correlation was not supported (*r* = 0.147) by the results.

### Validation of the side-by-side three-chamber strategy

**Fig 5:**
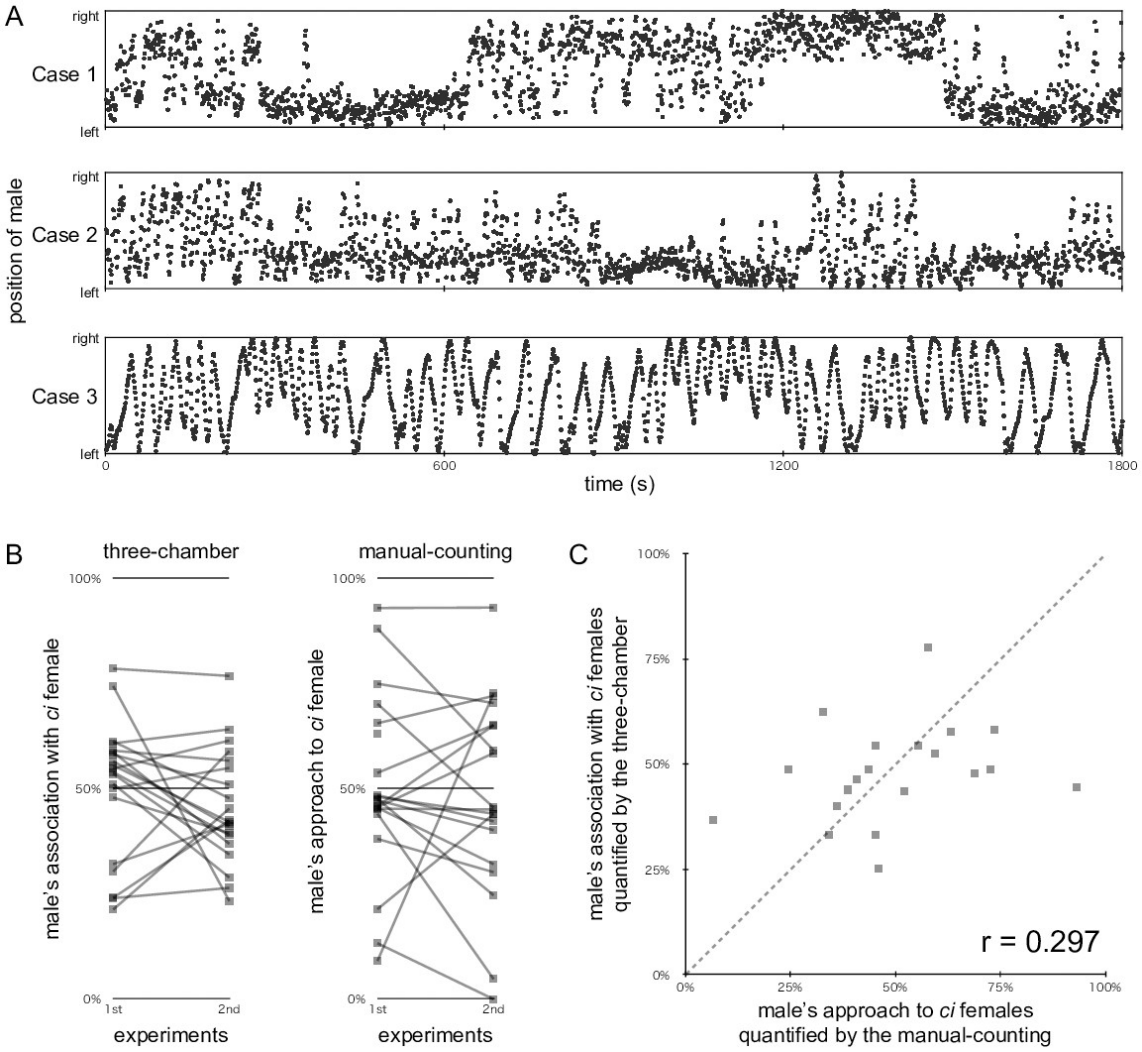
Assessment of the side-by-side three-chamber strategy. See Fig 4 for explanatory notes. (A) Examples of the movement of test males in front of the choice compartments (left and right). Males in Cases 1 and 2 showed interest in both or either mate choice female/s, respectively. The male in Case 3 had little interest in females but was still observed within 24 mm of the choice compartments for over 10 min; therefore, we included this datum in subsequent analyses. (B) Reproducibility of male preference in the three-chamber (left) or manual-counting (right) experiments. Test males (n = 20) were analysed using both the three-chamber and manual-counting strategies (i.e., each male was used in four experiments). A strongly biased association (e.g., <20% or >80%) was never observed in the three-chamber experiment (note that it was frequently [but non-reproducibly] observed in the face-to-face three-chamber experiment; Fig 4B) but was observed in the manual-counting experiment (as in Fig 4B). (C) Correlation analysis of the three-chamber and manual-counting results in (B).

Subsequently, we tested the validity of another (i.e., side-by-side) three-chamber apparatus (Fig 1, right). Fig 5A exemplifies the behaviour of the test male. The male in Case 1 was interested in both choice females (referred to as right and left), whereas the male in Case 2 preferred to associate with the female on the left. The male in Case 3 simply swam from side to side and seemed to have little interest in the females; however, this result, together with the remaining 39 results (i.e., 20 males × two experiments), was still valid according to our criterion (i.e., location within 24 mm from the choice compartments for 10 min or more).

An apparent difference between the results of the face-to-face and side-by-side apparatuses was that the ratio of association was never extreme (e.g., >80% or <20%) in the side-by-side apparatus (Fig 5B, left; see Fig 4B, left). Therefore, the results seemed to be somewhat reproducible between the first and second experiments with swapped female locations. However, some of these males exhibited a strong preference, as elucidated in the manual-counting experiment (Fig 5B, right). The comparison of the averaged manual-counting and three-chamber results did not support their correlation (Fig 5C; *r* = 0.297).

### Behaviour of the test females in the three-chamber apparatuses

**Fig 6:**
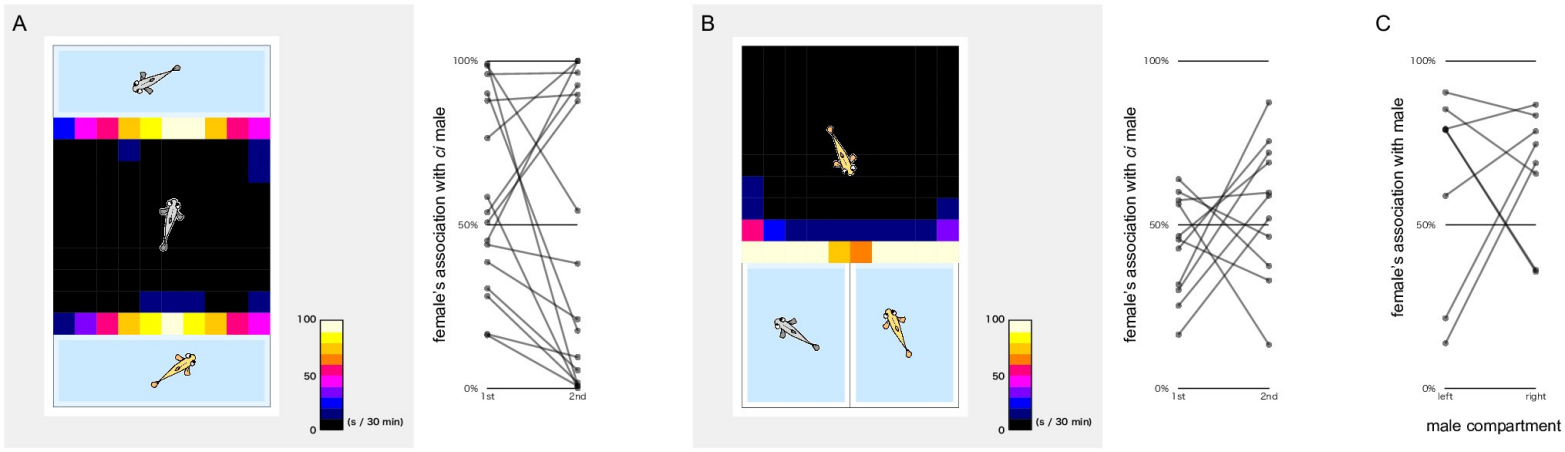
Behaviour of the test females in the face-to-face (A) and side-by-side (B) three-chamber experiments. Similar to the test males (Fig 3A), the females (n = 27 in total) spent most of the time in front of the choice compartments (left), but their association preferences were not reproducible (right). (C) Side-by-side three-chamber experiment with one empty compartment. Females (n = 8) largely preferred to associate with males. In four out of 16 experiments, however, the females preferred being in front of an empty compartment, which was never observed in males (Fig 3B).

We briefly assessed the behaviour of the test females in these three-chamber apparatuses by presenting males for them to choose. In both the face-to-face (n = 16; eight each for *ci* and Actb-SLα:GFP) and side-by-side (n = 11; all Actb-SLα:GFP) apparatuses, the females showed an apparent interest in, and spent most of the time in front of, the males (73.2% ± 3.2% and 91.4% ± 1.2%; Fig 6A and 6B, left). This active association of the females with the males was unexpected because the medaka females looked rather passive during mating; i.e., during insistent approaches from males (see Fig 2), the females seemed to make only a final decision regarding whether or not to spawn. Because such approaching females are rarely detected under a free-swimming condition, we hesitate to conclude that this active and persistent association between the females and the males in the three-chamber apparatuses (Fig 6A and 6B, left) was driven by sexual motivation.

Similar to the results obtained for the males (Figs 4B and 5B), a strongly biased preference was detected more frequently in the face-to-face location than in the side-by-side location (Fig 6A and 6B, right); however, this result was not reproducible between the duplicates with swapped male locations. We also assessed the behaviour of females (n = 8; all Actb-SLα:GFP) in the side-by-side three-chamber apparatus with one empty compartment (Fig 6C), as we did for males (Fig 3B). The overall association rate with the compartment containing a male was 64.7% ± 5.8%. However, some females occasionally preferred an empty compartment (in four out of 16 experiments), which was never observed in males.

Because the present data so far already suggested the invalidity of the three-chamber strategies (e.g., irreproducibility in swapped locations, inefficiency for detecting strong preferences, absence of correlation between the manual-counting and three-chamber results, and association with an empty compartment), we did not evaluate the sexual preference of the females under a free-swimming condition or assess the correlation of the results with those of the three-chamber experiments. For this purpose, we must prepare males that do not discriminate between and equally approach *ci* and Actb-SLα:GFP females (i.e., males plotted around 0.5 in Fig 2, bottom). Otherwise, *ci* males seldom approach Actb-SLα:GFP females and vice versa [16], which will cause a delay in spawning, regardless of the female’s preference, and bias the conclusion.

## Discussion

### Was the three-chamber strategy effective in previous medaka studies?

Males select females [23]. In fish, males of guppy, killifish, stickleback, cichlid and many other species prefer bigger females [24–29]; cichlid males prefer females with larger pelvic fins [30], halfbeak males prefer females with larger gravid spots [31], gobby males prefer females with an orange belly [32] and males of mosquitofish prefer conspecific females [19]. Many of those studies adopted the three-chamber strategy (including using digital images) for assessing the sexual preference of males. To the best of our knowledge, a positive correlation between association in the three-chamber test and actual mate choice has been suggested for some fish, including medaka [33, 34]. However, a careful re-examination of the medaka-related articles seemed to not necessarily support the conclusion, as explained below.

Grant et al. [33] demonstrated that medaka males spent more time with the larger females in a free-swimming condition (71% vs. 29%). Males also courted the largest females most frequently (61.3%, 30.4% and 8.3% toward large, intermediate and small females, respectively). In a face-to-face three-chamber apparatus of W30 × D60 (15 + 30 + 15) cm in which the water flow between the compartments was not fully restricted, 19 out of 25 males associated with larger (>17% longer in body length) females in a 1 × 30-min experiment; the authors concluded that males significantly preferred larger females based on a one-tailed binomial test (*P* = 0.0073). The actual association time or ratio, or the reproducibility of the results with a swapped location, were not available in that article.

Howard et al. [34] examined the association preference of medaka females in a face-to-face three-chamber apparatus of 40 L and showed that 40 out of 56 females preferred to associate with larger males (*P* < 0.001, Wilcoxon signed-rank test; an average association time of 16.0 ± 2.5 min in 2 × 10-min experiments), excluding an additional 32 females because of an association time of <60%. The females that preferred to associate with a larger male tended to mate with a larger (33% longer in body length) males (26/36); however, this choice might reflect a male–male competition, as indicated by the authors and other researchers [35].

We hesitate to conclude that the above results clearly demonstrated a qualitative/quantitative correlation between the free-swimming and three-chamber strategies. This is because, for example, the absence of swapping duplicates could reflect the position (instead of association) preferences (see Figs 4B and 6A), a 10-min experiment would detect an accidental association (see Fig 4A, Case 2; Grant et al. also stated that “Surprisingly, males did not appear discriminating about which female they courted first” [33], with which we also agree), and males compete each other (and guard a female) under a free-swimming condition, thus affecting the female’s choice [36]. Therefore, no previous studies seemed to demonstrate the validity of the three-chamber strategy in medaka.

### How should sexual preference be assessed in medaka?

**Fig 7:**
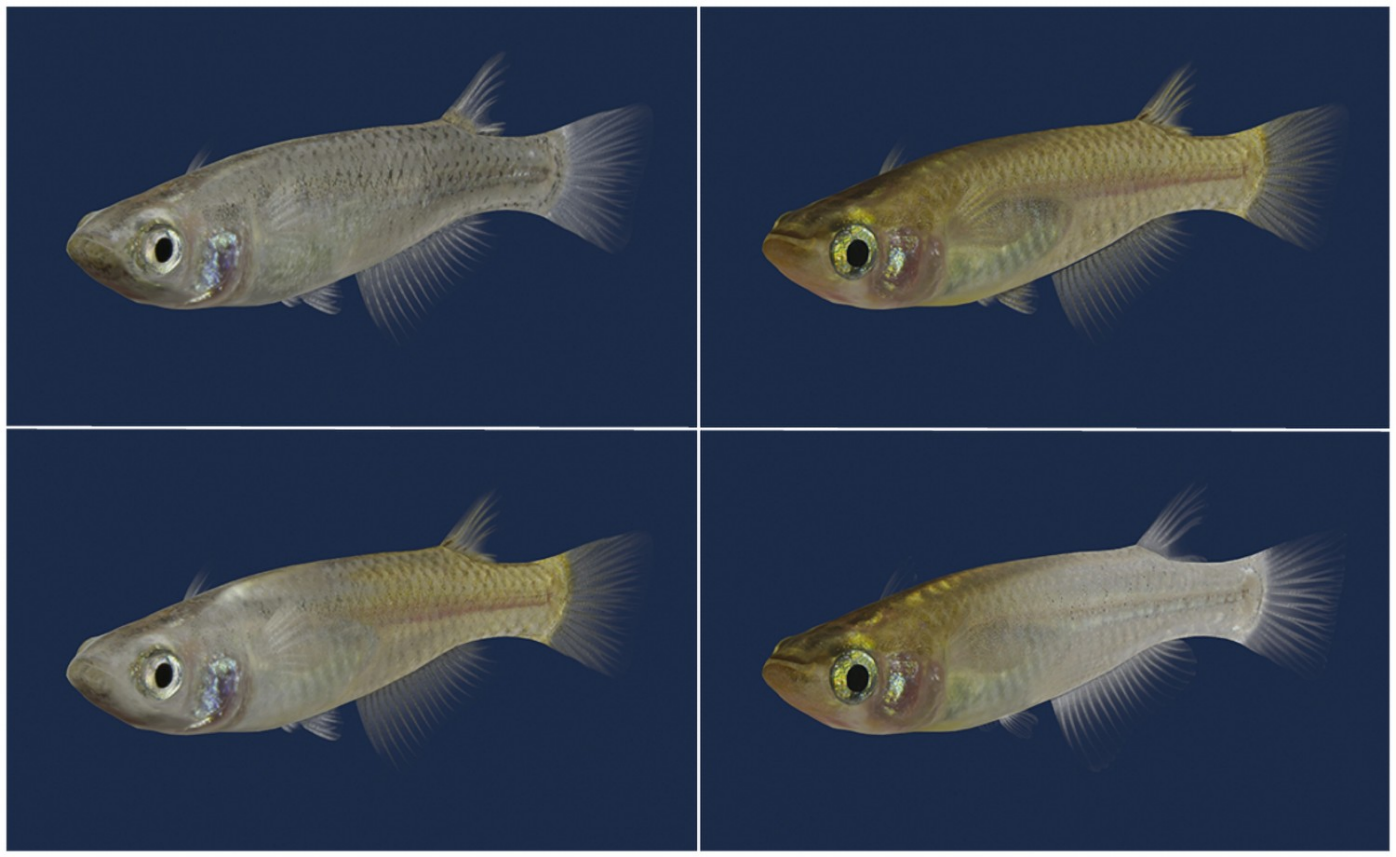
Three-dimensional computer graphics of *ci* (top left) and Actb-SLα:GFP (top right) fish. These colour variants are sexually isolated and mate assortatively [12–16]. These digital images could have been a unique tool for dissecting the colour-dependent mate choice, if the three-chamber strategy were proven to be valid in this study.

Before starting this study, we had been anticipating that the three-chamber strategy could expand our research opportunities for utilizing digital images (Fig 7), similar to the studies performed using other fish species [2–5]. Given the present results, however, neither the face-to-face nor the side-by-side strategy seemed to be effective for qualifying/quantifying the sexual preference of medaka males; i.e., medaka (both males and females) may persistently associate with inaccessible mates behind transparent walls (Figs 3A, 6A and 6B) for reasons other than sexual motivation.

Watanabe and his colleagues carried out some interesting experiments using digital images; e.g., the naturalness of the shape or motion of a 3D image is important for social attraction in medaka [37], but the biological motion of six dots was sufficient for the attraction [38] and a moving dot with pink noise could induce a predation behaviour [39]. Medaka is a shoaling species and some studies investigated shoaling preference using real fish [10, 40]. Generally, a three-chamber test could assess the tendency to approach and interact with conspecifics, which is the first and essential step in various social behaviours, not only mating, but also shoaling and fighting [41, 42]. Thus, although they are ready for mating, medaka in the three-chamber apparatuses might associate with mates for reasons other than a reproductive purpose. It is apparent that the three-chamber strategy is not always appropriate for assessing the sexual preference of animals. Ideally, we should quantify mating behaviours by observing free-moving animals in a semi-natural condition.

Further optimization (e.g., the size, shape or colour of the apparatus; the protocol of the experiment [e.g., acclimation/observation/interval period]; or the definition of the association [e.g., zone area, distance from mates or direction of the body]) may render the three-chamber strategies effective. We also tested another face-to-face three-chamber apparatus (W150 × D210 [30 + 150 + 30] mm), but obtained preliminary results that were not promising (data not shown). Manual counting of male approaches in a three-chamber apparatus could somehow reproduce the sexual preferences of *ci* and Actb-SLα:GFP individuals [12], but the total number of approaches was greatly reduced, and the strategy was not at all labour saving.

At present, the most reliable strategy for quantifying the sexual preference of medaka remains manual observation; i.e., counting the number of approaches, dances or copulation attempts by males [10–16], and measuring the duration or counting the number of approaches received from males (or rejected by females) until spawning for females [8, 9, 11, 12, 35, 36, 43, 44]. To expand research opportunities (e.g., utilization of digital images; Fig 7), a technical innovation is awaited.

## Conclusions

The three-chamber strategy tested here was not valid for medaka mate-choice studies. Although negative, this result demonstrated the difficulty of applying the three-chamber strategy even to a species that spawns every day, which should be widely noted by ethologists interested in mate choice. Medaka might be a mere exception of no importance, or a similar problem may lie dormant among other species.

## Supporting information

S1 Data (XLSX)
